# The Root Extract of *Rosa multiflora* Ameliorates Nonalcoholic Steatohepatitis Development via Blockade of De Novo Lipogenesis and Inflammation

**DOI:** 10.3390/cimb46060351

**Published:** 2024-06-12

**Authors:** Nam-Hee Kim, Seung-Jin Lee, Kyeong-Jin Lee, Ae Ri Song, Hyun-Je Park, Jong Soo Kang, Joo Young Cha, Yong-Hyun Han

**Affiliations:** 1 Laboratory of Pathology and Physiology, College of Pharmacy, Kangwon National University, Chuncheon 24341, Republic of Korea; 9468993@naver.com (N.-H.K.); berry7050@naver.com (S.-J.L.); rudwls1134@naver.com (K.-J.L.); 2 Yuhan Care Co., Ltd., Yuhan Care R&D Center, Yongin-si 17084, Republic of Korea; aeri.song@yuhancare.com (A.R.S.); hjpark@yuhancare.com (H.-J.P.); jskang@yuhancare.com (J.S.K.); 3 Multidimensional Genomics Research Center, Kangwon National University, Chuncheon 24341, Republic of Korea

**Keywords:** NASH, YC-1102, steatosis, hepatocyte, macrophage

## Abstract

Nonalcoholic steatohepatitis (NASH) is characterized by severe inflammation and fibrosis due to an excessive accumulation of triglycerides (TGs) in the liver with a dysregulated de novo lipogenesis (DNL) pathway. In this study, we aimed to evaluate the effectiveness of YC-1102, an extract obtained from the roots of *Rosa multiflora*, as a nutritional supplement in a diet-induced NASH mouse model. C57BL/6 wild-type mice were fed a fructose, palmitate, and cholesterol (FPC)-containing diet for 16 weeks to induce experimental NASH. A daily oral gavage of YC-1102 and obetichoic acid (OCA) was conducted for 9 weeks. After sacrifice, disease parameters related to hepatic lipids, inflammation, and fibrosis were evaluated. The treatment with YC-1102 significantly decreased the liver/body weight ratio, epididymal fat weight, and plasma ALT and AST levels, which are indicators of NASH injuries. YC-1102 attenuated hepatic lipid accumulation by inhibiting the transcription of DNL genes in the livers exhibiting NASH. Additionally, we found that YC-1102 blocked the development of hepatic inflammation and fibrosis by directly disturbing macrophage activation, resulting in an amelioration of hepatic fibrosis. Our findings suggest that YC-1102 could ameliorate NASH progression by inhibiting uncontrolled DNL and inflammation.

## 1. Introduction

Nonalcoholic fatty liver disease (NAFLD) is defined as an ectopic accumulation of triglycerides (TGs) in the liver due to increased de novo lipogenesis (DNL) [1]. Nonalcoholic steatohepatitis (NASH) is type of NAFLD that involves extensive hepatic inflammation and fibrosis caused by an excessive accumulation of fat. Although the prevalence of patients suffering with NASH is increasing, no approved clinical therapies to treat it have yet been developed [2]. Hepatic de novo lipogenesis involves the synthesis of fatty acids from acetyl-CoA subunits and is an essential biosynthetic pathway for liver homeostasis. DNL is a metabolic process in which, primarily, carbohydrate sources are converted to fatty acids [3]. Glucose derived from carbohydrates undergoes glycolysis and enters the tricarboxylic acid (TCA) cycle, producing citrate. ATP-citrate lyase (ACLY) produces acetyl-CoA from this citrate. Acetyl-CoA is converted to malonyl-CoA by acetyl-CoA carboxylase 1 (ACC1) and fatty acid synthase (FASN) converts malonyl-CoA to palmitic acid. Palmitate is converted into diverse fatty acids via catalytic desaturation by stearoyl-CoA desaturase-1 (SCD1). The synthesized fatty acids are esterified to form TG [4,5]. DNL inhibitors such as ACC and SCD1 are under clinical trials for preventing NASH progression [6]. The transcriptional expression of these DNL genes is mainly regulated by metabolic transcription factors such as peroxisome proliferator-activated receptor **γ** (PPAR**γ**) and sterol regulatory element-binding protein 1 (SREBP1), which play important roles in fatty liver development [7]. PPAR**γ** and SREBP1 enhance the transcriptional activity of the promoters and enhancers of DNL genes, indicating that an imbalance of PPAR**γ** and SREBP1 can exacerbate NASH development.

As NAFLD progresses with an excessive accumulation of hepatic lipids, the pro-inflammatory activation of liver macrophages is triggered by pathogen-associated molecular patterns (PAMPs) and damage-associated molecular patterns (DAMPs) [8]. In particular, the lipotoxic overburden results in dead hepatocytes, which release DAMPs and harmful lipid mediators that promote macrophage activation [9]. Gut-derived bacterial endotoxins (lipopolysaccharides; LPSs) that invade through high-fat-mediated intestinal leakage are increased under NASH conditions, and then trigger Toll-like receptor 4 (TLR4)-mediated production of pro-inflammatory cytokines in macrophages [10]. Activated liver macrophages initiate the fibrotic activation of hepatic stellate cells (HSCs) via the release of various cytokines and chemokines such as transforming growth factor β (TGFβ), tumor necrosis factor α (TNFα), platelet-derived growth factor, and interleukin-18 (IL-18) [11,12]. Additionally, DAMPs derived from injured hepatocytes lead to skewing quiescent HSCs to the activated state [13]. Therefore, suppressing the catastrophic cascade of hepatic steatosis and macrophage activation could delay the development of severe liver fibrosis.

*Rosa multiflora* Thunb is a plant in the Rosaceae family and is distributed in East Asia, including in Korea. The roots of *R. multiflora* have been reported to contain tannins including novel stereoisomers as well as catechin (CAT), and procyanidin B-3 (ProB3) [14]. Furthermore, previous studies have reported that the roots of *R. multiflora* have strong anti-inflammatory effects in mouse models of atopic dermatitis [15]. Interestingly, *R. multiflora* root showed beneficial effects in reducing cholesterol due to improved bile acid and lipid metabolism in high-fat diet-fed mice [16]. Previously, our group collected YC-1102, an extract of *R. multiflora* roots, and found that it effectively reduced high-fat-induced adipogenesis, indicating that YC-1102 has anti-obesity effects and the potential to reduce hepatic fat accumulation [17]. Therefore, we aimed to further investigate whether YC-1102 had protective effects on hepatic steatosis, inflammation, and fibrosis in a diet-induced NASH mouse model to investigate its potential use as a nutritional supplement to ameliorate NASH progression.

## 2. Materials and Methods

### 2.1. Preparation of Rosa multiflora (YC-1102)

The test material YC-1102 was prepared through the extraction of the roots of *R. multiflora* harvested at the Goesan-gun herbal medicine farm in July 2021, which grows over 3 years naturally in South Korea. *R. multiflora* roots were extracted at 60 °C for 5 h using 70% ethanol at 10.7 (*w*/*v*) based on the dry weight. The extract was then concentrated with a 1 μm filter to 20 ± 5 °Brix. Subsequently, the concentrated YC-1102 was spray-dried (MH-8, mehyun engineering Ltd., Gyeonggi-do, Republic of Korea) with an inlet temperature of 170 ± 5 °C and an outlet temperature of 100 ± 5 °C [18]. The YC-1102 solution was standardized through the HPLC analysis of rosamultin content [Supplementary Figure S1]. The ACQUITY UPLC H class (Waters, Milford, MA, USA)—used to conduct the HPLC analysis—consists of a quaternary pump (ACQUITY QSM) and a UV/vis detector (ACQUITY UPLC TUV) that can detect an output signal at a 205 nm wavelength. An amount of 100 mg of YC-1102 was put into a 10 mL volumetric flask and dissolved through ultrasonication with 10 mL 50% methanol for 5 min. Afterward, the solution was filtered with a 0.45 μm syringe filter (Hyundal Micro Co., Ltd., Seoul, Republic of Korea) and used as the test solution. Rosamultin standards were also dissolved in 50% MeOH for sonication and filtration. As expected, 10.3 mg of rosamultin was detected in 1 g of Rosa multiflora root extract, which is about 1.03%. Standard solutions were prepared at concentrations of 25, 50, 100, 250, and 500 μg/mL. YC-1102 was separated chromatographically using a Zorbax Eclipse XDB-C18 column (4.6 mm × 150 mm, 5 μm, Agilent Technologies, Palo Alto, CA, USA) with a column temperature of 30 °C. The isocratic mobile phase consisted of water/methanol/phosphoric acid (32.9:66.9:0.2, *v*/*v*/*v*) delivered at a flow rate of 1.00 mL/min with an injection volume of 10 μL. The HPLC analysis was repeated three times within a week to measure the rosamultin content in YC-1102.

### 2.2. Animal Experiment

Eight-week-old C57BL/6 male mice (*n* = 37) were obtained from Orient Bio Inc. (Seongnam, Republic of Korea). The mice were divided into two experimental groups: a chow diet group (*n* = 5) and a NASH diet group (*n* = 32), fed a fructose, palmitate, and cholesterol (FPC)-containing diet (Envigo, Huntingdon, UK) with sugar water (23.1 g fructose + 18.9 g glucose in 1 L of autoclaved tap water) [19]. The mice were fed these diets for 16 weeks. After feeding the mice with the FPC diet for 7 weeks (*n* = 32), the diet was supplemented with a vehicle (*n* = 8), 100 mg/kg of YC-1102 (*n* = 8), 200 mg/kg of YC-1102 (*n* = 8), or 30 mg/kg of obeticholic acid (OCA) (*n* = 8), which was administered via daily oral gavage for 9 weeks. According to a recent report, oral administration of YC-1102 at doses of 100, 150, and 200 mg/kg for 8 weeks inhibits adipogenesis and adipocyte differentiation in obese mice induced by a high-fat diet [17]. Based on these findings, the dose for this study was determined. YC-1102 was diluted in sterile phosphate-buffered saline (PBS) and OCA (MedChemExpress, Monmouth Junction, NJ, USA) was diluted in 0.5% carboxymethyl cellulose with 0.01% Tween-80 in distilled water. CO_2_ euthanasia was conducted on the mice to obtain blood from the inferior vena cava and tissues with careful checks for signs of mouse stress following animal guidelines using the gradual fill method. After abdominal laparotomy, the epididymal fat and liver tissues were isolated and fixed in 4% paraformaldehyde and stored at −80 °C. The left lobe of the liver was used for mRNA analysis and hepatic TG measurement. All the experiments were performed in a blinded and randomized fashion according to the guidelines of the Animal Care and Use Committee of Kangwon National University (KW-IACUC-220412-6 and KW-IACUC-210923-1).

### 2.3. Histology (Oil Red O, Sirius Red, and Immunohistochemistry)

Sections of liver tissue were fixed in 4% paraformaldehyde (Biosesang, Seoul, Republic of Korea) and embedded in paraffin. To observe hepatic fibrosis, liver sections (5 μm of thickness) were stained using Picro-Sirius Red (Abcam, Cambridge, UK). In addition, sections were stained with Hematoxylin and Eosin (H&E, Sigma-Aldrich, St. Louis, MO, USA). For immunohistochemical staining, anti-F4/80 (Abcam) was used with Hematoxylin QS and peroxidase substrate (Vector laboratories, Burlingame, CA, USA). Oil red O (Abcam) was used to stain cryosections of the liver tissue.

### 2.4. Real-Time PCR

Total RNA was extracted from frozen liver tissues using the RNeasy mini kit with Tissue Lyser II (Qiagen, Hilden, Germany) according to the manufacturer’s protocol. After isolating the primary cells, total RNA was extracted using the XENOPURE Small RNA Purification kit (Xenohelix, Incheon, Republic of Korea). The quality and concentration of the extracted total RNA were analyzed with a UV/Vis Nabi Spectrophotometer (MicroDigital, Seongnam, Republic of Korea). cDNA was generated through the reverse transcription of purified RNA using a high-capacity cDNA reverse transcription kit (Thermo Fisher Scientific, Waltham, MA, USA). Real-time PCR experiments were performed using the ABI StepOnePlus™ Real-Time PCR system and SYBR^®^ Green PCR Master Mix (Applied Biosystems, Foster City, CA, USA) to quantify the amplification of PCR products using specific primers, which are listed in Table 1. The relative mRNA levels of target genes were estimated with the eq. 2-Ct (ΔCt = Ct of the target gene minus Ct of 18S rRNA). The relative changes in gene transcription were calculated based on a level of 1 for the control group.

### 2.5. Plasma ALT, AST, and TG

After systemic venous blood was collected from the inferior vena cava, plasma was separated after centrifugation at 3400× *g* for 10 min at 4 °C. Then, plasma alanine transaminase (ALT), aspartate transaminase (AST), and TGs were measured using corresponding chips (Fujifilm, Sendai, Japan). They were measured with a Fuji DRI-CHEM 3500s serum biochemistry analyzer (Fujifilm).

### 2.6. Hepatic TG Measurement

Frozen liver tissues were homogenized in RIPA lysis buffer containing protease inhibitor cocktail (Sigma-Aldrich) and incubated on ice for 30 min. After centrifugation at 18,400× *g* for 10 min, the supernatant containing lipids was collected and the TG levels were measured using the EnzyChrom™ Triglyceride Assay Kit (BioAssay Systems, Hayward, CA, USA), following the manufacturer’s recommendation.

### 2.7. Cell Isolation and Culture

Primary hepatocytes were isolated from the livers of C57BL/6 mice. Hanks’ Balanced Salt Solution (Sigma-Aldrich) containing collagenase IV (Sigma-Aldrich) was perfused. The livers were suspended in Medium 199 media (Sigma-Aldrich) containing 10% Fetal Bovine Serum (FBS), 1% penicillin/streptomycin, 23 mM HEPES, and 10 nM dexamethasone [20]. The cells were filtered with a 100 μm cell strainer and centrifuged at 50× *g* for 2 min. Medium 199 was added to the cell pellets, and then they were stacked using Percoll (GE Healthcare, Madison, WI, USA) and centrifuged at 330× *g* for 3 min to remove dead hepatocytes. After centrifugation at 50× *g* for 3 min, the supernatant containing HSCs was obtained and centrifuged again at 570× *g* for 5 min to collect the cell pellet. Cell pellets were layered on 11.5% OptiPrep (Sigma-Aldrich) and separated through centrifugation at 1400× *g* for 15 min. DMEM medium containing 10% FBS and 1% penicillin/streptomycin was used to culture primary HSCs. On day 4, HSCs were activated through treatment with 5 ng/mL of TGF-β (R&D system, Minneapolis, MN, USA) and YC-1102 at concentrations of 20 μg/mL and 50 μg/mL overnight.

Bone marrow-derived macrophages (BMDMs) were isolated from bone marrow from the femur and tibias [21]. Monocytes were isolated and seeded in 24 wells with DMEM medium containing 10% FBS and 1% penicillin/streptomycin. Monocyte to macrophage differentiation was induced through treatment with 40 ng/mL of M-CSF (Peprotech, Rocky Hill, NJ, USA) for 3 days. On day 4, the samples were treated with YC-1102 at different concentrations overnight and then 100 ng/mL of LPS to induce an inflammatory response.

### 2.8. Statistics

All values are presented as the mean ± standard deviation (SD). All the graphs were produced and analyses were performed using GraphPad Prism 8.0.1 (La Jolla, CA, USA). Statistical analysis was conducted using a one-way ANOVA with Bonferroni’s post hoc test for multiple comparisons. *p* < 0.05, *p* < 0.01 and *p* < 0.001 were considered statistically significant.

## 3. Results

### 3.1. YC-1102 Ameliorates Symptoms of FPC Diet-Induced NASH

**Figure 1:**
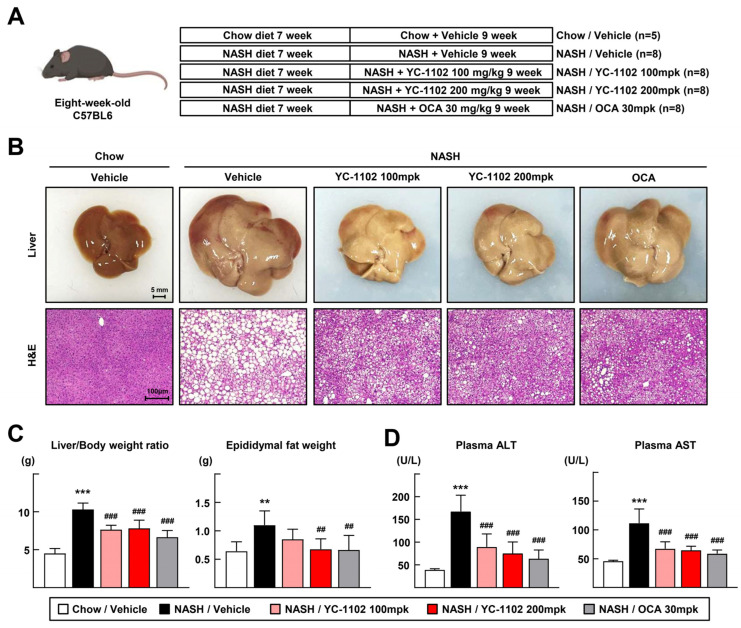
Treatment with YC-1102 ameliorates the progression of FPC diet-induced NASH. (**A**) Experimental design of YC-1102 treatment in FPC-induced NASH mouse model. (**B**) Representative images of livers and H&E staining for sectioned liver. (**C**) Liver/body weight ratio (**left**) and epididymal fat weight (**right**) at the end of experiments. (**D**) Activities of plasma ALT and AST were measured. *n* = 5 for chow and *n* = 8 for NASH. ** *p* < 0.01 and *** *p* < 0.001 vs. chow with vehicle; ## *p* < 0.01 and ### *p* < 0.001 vs. NASH with vehicle. The data represent mean ± SD. Statistical analyses were performed using one-way ANOVA with Bonferroni’s post hoc test.

We obtained extracts of YC-1102 and tested whether YC-1102 alleviated liver injuries in a FPC-containing-diet-induced NASH model after HPLC analysis to determine the components of YC-1102 containing rosamutin. As a result of the HPLC analysis of YC-1102, rosamultin was detected as the standard component because it produced a single peak completely separated without the influence of interfering components [Supplementary Figure S1]. The detection of the other components was difficult because of the peak interactions and low content. NASH was induced by feeding the mice an FPC diet for 16 weeks. At 9 weeks of diet feeding, the vehicle, 100 mpk of YC-1102 (100 mg/kg), 200 mpk of YC-1102 (200 mg/kg), or OCA (30 mg/kg) was administered daily [Figure 1A]. The OCA-treated group was employed as a positive comparative control because OCA treatment has shown protective effects against NASH progression [22]. YC-1102 suppressed the development of an inflamed and enlarged NASH liver, and the H&E staining confirmed that hepatocyte ballooning and fat droplets were reduced after YC-1102 treatment [Figure 1B]. The liver/body weight ratio significantly decreased in the YC-1102-treated groups, and the epididymal fat weight was also reduced by YC-1102 administration [Figure 1C]. The plasma activities of ALT and AST, indicators of liver damage, were reduced in the YC-1102- and OCA-treated groups, indicating that YC-1102 effectively prevents FPC diet-induced NASH injuries, similarly to OCA [Figure 1D].

### 3.2. De Novo Lipogenesis-Induced Hepatic Steatosis Is Inhibited by YC-1102 Treatment

**Figure 2:**
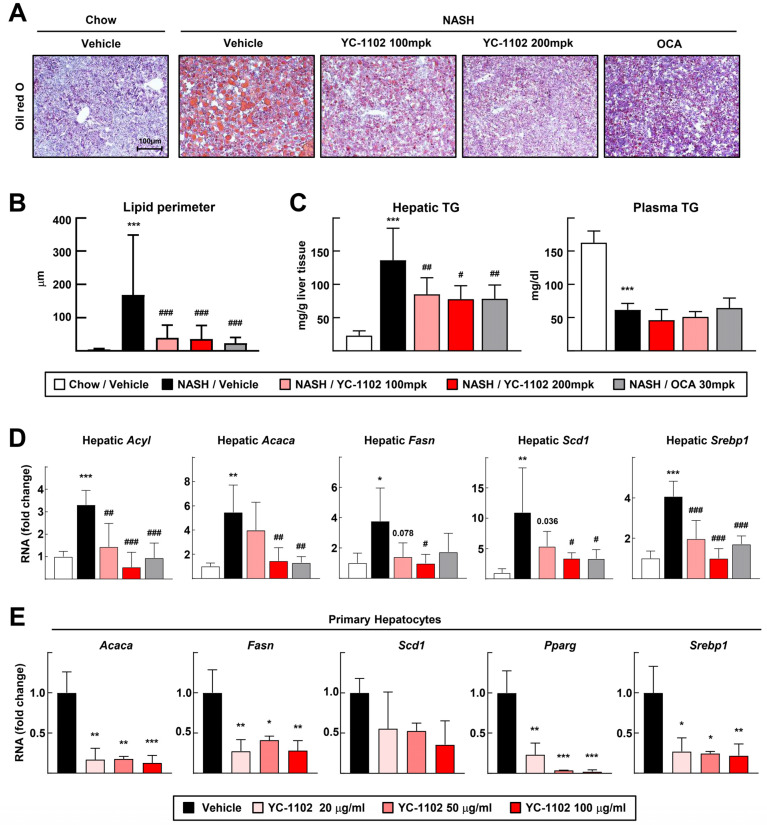
YC-1102 reduces hepatic lipid accumulation via transcriptional downregulation of genes involved in de novo lipogenesis. (**A**) Representative images of Oil red O staining for sectioned livers. (**B**) Lipid perimeters in Oil red O staining were measured. (**C**) Hepatic and plasma TG levels were measured. *n* = 5 for chow and *n* = 8 for NASH. (**D**) Hepatic mRNA transcriptions of *Acyl*, *Acaca*, *Fasn*, *Scd1*, and *Srebp1* were analyzed using qRT-PCR. *n* = 5 for each group. * *p* < 0.05, ** *p* < 0.01, and *** *p* < 0.001 vs. chow with vehicle; # *p* < 0.05, ## *p* < 0.01 and ### *p* < 0.001 vs. NASH with vehicle. (**E**) Primary hepatocytes were treated with YC-1102 after oleic acid-incubated lipid accumulation. mRNA transcriptions of *Acaca*, *Fasn*, *Scd1*, *Pparg*, and *Srebp1* in primary hepatocytes were analyzed using qRT-PCR. *n* = 3 for each group. * *p* < 0.05, ** *p* < 0.01, and *** *p* < 0.001 vs. vehicle. The data represent mean ± SD. Statistical analyses were performed using one-way ANOVA with Bonferroni’s post hoc test.

Next, we analyzed the effects of YC-1102 on changes in the hepatic lipid contents. The Oil red O staining of the liver sections showed that YC-1102 reduced the accumulation of fat in the livers, and the size of the lipid droplets was remarkably decreased by the oral gavage of YC-1102 [Figure 2A,B]. YC-1102 significantly decreased the hepatic TG levels, but did not alter the plasma TG levels [Figure 2C]. In addition, we measured the transcription of DNL genes such as *Acyl*, *Acaca*, *Fasn*, and *Scd1.* The mRNA transcriptions of these DNL genes was dose-dependently downregulated by YC-1102 treatment [Figure 2D]. We also conducted in vitro experiments by isolating primary hepatocytes. Consistently, YC-1102 largely decreased the transcription levels of the DNL genes [Figure 2E]. We further found that YC-1102 repressed the mRNA levels of transcription factors of DNL markers including *SREBP1* and *PPARγ* in the liver and hepatocytes [Figure 2D,E]. These data suggest that the YC-1102 treatment reduced the transcription of DNL genes by suppressing transcription factors, resulting in an attenuation of hepatic lipid accumulation.

### 3.3. YC-1102 Attenuates Hepatic Inflammation through Suppression of Macrophage Activation

**Figure 3:**
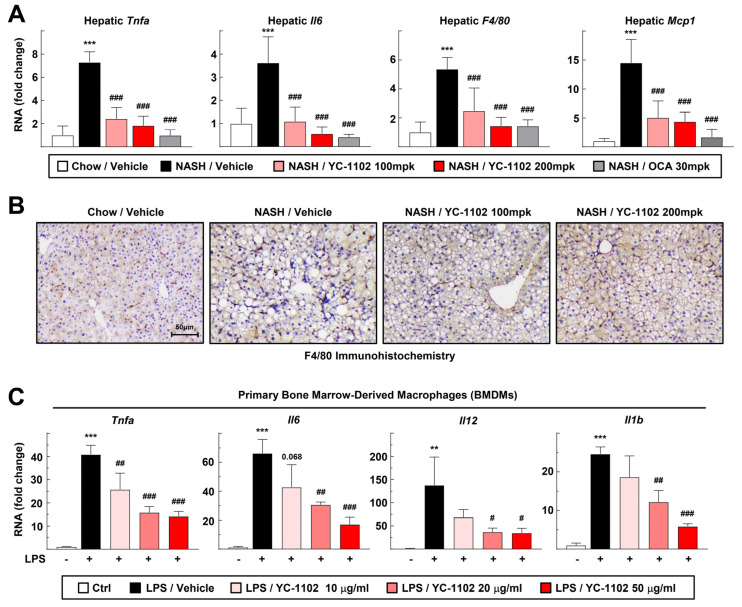
Anti-inflammatory effects of YC-1102 via direct suppression of macrophages. (**A**) Hepatic mRNA transcriptions of *Tnfa*, *Il6*, *F4/80,* and *Mcp1* were analyzed using qRT-PCR. *n* = 5 for each group. *** *p* < 0.001 vs. chow with vehicle; ### *p* < 0.001 vs. NASH with vehicle. (**B**) Representative images of immunohistochemical staining of F4/80 for sectioned livers. (**C**) Primary BMDMs were stimulated with LPS after YC-1102 treatment. mRNA transcriptions of *Tnfa*, *Il6*, *Il12*, and *Il1b* in primary BMDMs were analyzed using qRT-PCR. *n* = 3 for each group. ** *p* < 0.01 and *** *p* < 0.001 versus Ctrl; # *p* < 0.05, ## *p* < 0.01, and ### *p* < 0.001 vs. LPS with vehicle. The data represent mean ± SD. Statistical analyses were performed using one-way ANOVA with Bonferroni’s post hoc test.

We further investigated whether YC-1102 also affected hepatic inflammation. The transcription of hepatic inflammation markers such as *Tnfa*, *Il6*, *F4/80*, and *Mcp1* in the liver tissue was significantly decreased by the YC-1102 treatment [Figure 3A]. In addition, the staining of liver macrophages through immunohistochemistry showed a reduction in macrophage cells in the YC-1102-treated group [Figure 3B]. We isolated the primary BMDMs to test the direct anti-inflammatory effects of YC-1102 on the macrophage cells. Interestingly, YC-1102 directly downregulated the transcription of inflammatory cytokines, including *Tnfa*, *Il6*, *Il12*, and *Il1b*, in the BMDMs in a dose-dependent manner [Figure 3C]. These results indicate that YC-1102 suppresses hepatic inflammation by inhibiting hepatic DNL and macrophage activation.

### 3.4. YC-1102 Suppresses Hepatic Fibrosis in FPC Diet-Induced NASH

**Figure 4:**
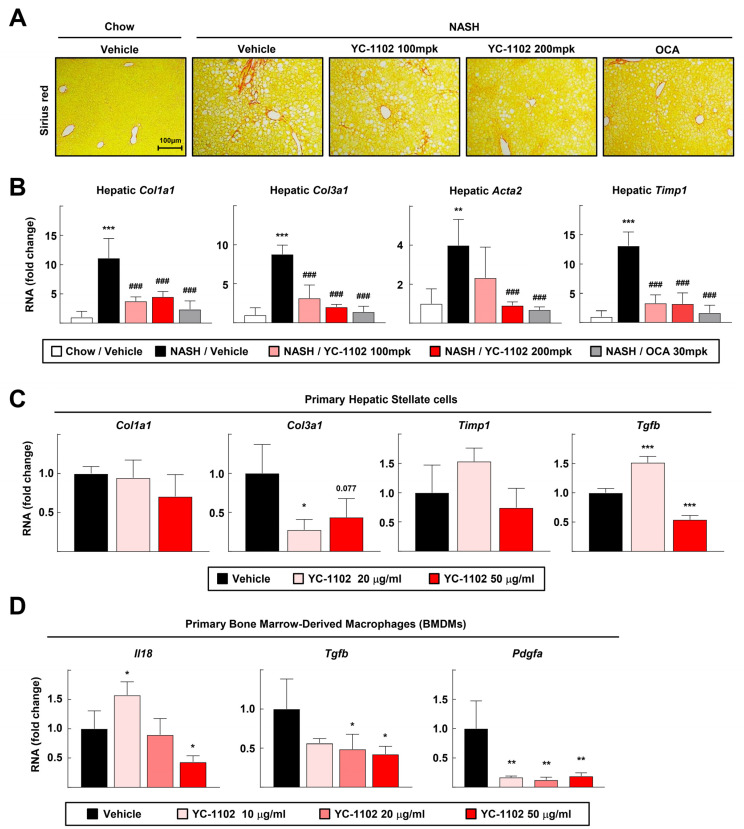
Anti-fibrotic effects of YC-1102 in the liver. (**A**) Representative images of Sirius Red staining of liver tissue. (**B**) Hepatic mRNA transcriptions of *Col1a1*, *Col3a1*, *Acta2*, and *Timp1* were analyzed using qRT-PCR. *n* = 5 for each group. ** *p* < 0.01 and *** *p* < 0.001 vs. chow with vehicle; ### *p* < 0.001 versus NASH with vehicle. (**C**) Primary hepatic stellate cells (HSCs) activated through TGFβ stimulation were treated with YC-1102. mRNA transcriptions of *Col1a1*, *Col3a1*, *Timp1*, and *Tgfb* in primary HSCs were analyzed using qRT-PCR. *n* = 4 for each group. (**D**) Primary BMDMs were stimulated with LPS after YC-1102 treatment. mRNA transcriptions of *Il18*, *Tgfb*, and *Pdgfa* in primary BMDMs were analyzed using qRT-PCR. *n* = 4 for each group. * *p* < 0.05, ** *p* < 0.01, and *** *p* < 0.001 vs. vehicle. The data represent mean ± SD. Statistical analyses were performed using one-way ANOVA with Bonferroni’s post hoc test.

In NASH development, quiescent HSCs are activated to induce hepatic fibrosis when liver cell injuries are exacerbated as a result of excessive fat accumulation and inflammation [13]. Previous data revealed that YC-1102 notably reduced the hepatic lipid content and inflammation. Therefore, we investigated whether YC-1102 alleviated liver fibrosis. The Picro-Sirius Red staining of the liver sections consistently showed that collagen deposition was alleviated in the YC-1102- and OCA-treated group [Figure 4A]. In addition, the transcript levels of liver fibrosis-related genes such as collagen type I alpha 1 chain (*Col1a1*) and collagen type III alpha 1 chain (*Col3a1*) were significantly downregulated by YC-1102, indicating that YC-1102 could inhibit NASH-induced liver fibrosis [Figure 4B]. However, YC-1102 did not effectively decrease the transcripts of fibrotic genes in primary HSCs, leading to speculation that the attenuation of YC-1102-mediated liver fibrosis might be hepatocyte- and macrophage-dependent [Figure 4C]. Indeed, we confirmed that YC-1102 dramatically suppressed the mRNA transcriptions of stellate cell-activating cytokines such as *Il18*, *Tgfb*, and *Pdgfa* [Figure 4D]. Altogether, we suggest that treatment with YC-1102 can prevent fibrotic progression in diet-induced NASH via a blockade of macrophage activation.

## 4. Discussion

The global burden of NASH is currently growing, and the progression of NASH can increase cardiovascular mortality [23]. An excessive burden of steatosis leads to the development of hepatic inflammation and fibrosis, resulting in catastrophic liver cirrhosis [24]. Steatosis is an essential step in the development of NASH because the excessive accumulation of hepatic lipids leads to the activation of inflammation in liver macrophages [25]. Accordingly, preventing hepatic fat accumulation through the regulation of aberrant fatty acid metabolism would be a promising therapeutic strategy. Indeed, drugs targeting the inhibition of DNL proteins such as Firsocostat (an ACC inhibitor) and Aramchol (an SCD inhibitor) are currently under clinical trials [26]. In this study, we found that extracts of *R. multiflora* root show protective effects on NASH development via suppressing DNL activation, and underlying YC-1102 might be a therapeutic option to ameliorate NASH diseases.

In this study, we found that YC-1102 was effective in alleviating diet-induced NASH symptoms. *Rosa multiflora,* belonging to the *Rosaceae* family, is a deciduous shrub widely distributed in the mountains of East Asia including in Korea, Japan, and China. Triterpenoid tormentic acid and glycoside rosamultin are abundantly found in the roots of *R. multiflora* and show anti-inflammatory effects [27]. Tormentic acid is classified as a pentacyclic triterpene and extensive evidence has been found regarding its ability to reduce aspects of NASH pathogenesis such as steatosis, oxidative stress, inflammation, and fibrosis [28]. But in our HPLC experiments, interference peaks were detected in the peaks of euscaphic acid and tormentic acid. We suggest that tormentic acid in YC-1102 could also contribute to improving NASH diseases. Glycoside rosamultin is abundant in the root of *R. multiflora* and shows lipid-lowering effects [27]. The YC-1102 solution was confirmed to contain rosamultin through an HPLC analysis and standardization [Supplementary Figure S1] [16]. According to a recent study, rosamultin significantly reduces the mRNA levels of key genes related to adipogenesis and fat synthesis, such as *Cebpα*, *Pparγ*, *Srebp1c*, *Fas*, *Acc*, and *Ap2*, effectively inhibiting the accumulation of triglycerides in adipocytes. Rosamultin has also been reported to be effective in lowering the triglyceride levels in adipocyte cell lines, resulting in a reduction in fat synthesis [29]. In this study, specific components of *R. multiflora* root extract such as rosamultin were found to have lipid-reducing effects by modulating various metabolic regulators, resulting in an attenuation of NASH progression.

The transcriptional activation of DNL is regulated by the transcription factor SREBP1, which binds to the DNA of sterol regulatory elements (SREs) and increases the expression of DNL genes, resulting in increased TG accumulation in the liver. Additionally, PPARγ, a member of the nuclear hormone receptor superfamily, promotes fatty acid uptake and TG synthesis [30,31,32]. Our results showed that treatment with YC-1102 reduced the transcriptional expression of *Srebp1* and *Pparg*, leading to a reduction in the mRNA levels of downstream DNL genes such as *Acyl*, *Acaca*, *Fasn*, and *Scd1*. Therefore, NASH progression was ameliorated because YC-1102 blocks hepatic lipid accumulation by interfering with transcription factor-induced activation of the DNL pathway. The details of the molecular mechanisms by which components of YC-1102 decrease the transcription of *Srebp1* and *Pparg* should be further investigated.

DAMPs released from damaged hepatocytes and gut-derived PAMPs stimulate hepatic macrophages to secrete proinflammatory cytokines and trigger NASH-induced hepatic inflammation [8]. Furthermore, quiescent HSCs are activated by the fibrotic mediators released from inflammatory macrophages, driving hepatic collagen accumulation [13]. We confirmed that treatment with YC-1102 suppressed liver inflammation and directly repressed the inflammatory activation of macrophages [Figure 3]. Furthermore, YC-1102 attenuated liver fibrosis by inhibiting the mRNA transcriptions of fibrotic mediators in macrophages [Figure 4]. Nuclear factor-κB (NF-κB) is a major transcription factor that induces the inflammatory activation of macrophages [33]. Toll-like receptors are involved in PAMP-induced changes in macrophages that tip them toward a pro-inflammatory state [34]. YC-1102 might constrain the transcriptional activity of NF-κB and the signaling pathway of TLRs. Therefore, further investigation to find specific components of YC-1102 that modulate inflammatory signaling pathways will be of interest for treating various inflammatory diseases.

## 5. Conclusions

According to our study, for the first time, we found that the daily administration of YC-1102 reduced the liver/body weight ratio and plasma liver injury parameters such as ALT and AST levels in mice with NASH (Figure 1). YC-1102 downregulated the transcription of DNL genes in the diet-induced NASH livers, and a similar result was observed in the primary hepatocytes (Figure 2). It was further confirmed that YC-1102 blocked the transcriptional activation of inflammatory cytokine genes in macrophages (Figure 3). In addition, the YC-1102 treatment prevented fibrotic progression by decreasing the mRNA transcription of HSC-activating cytokines derived from macrophages (Figure 4). These findings highlight the novel anti-NASH effects of YC-1102 in directly regulating macrophages and hepatic stellate cells. In conclusion, we demonstrated that YC-1102 could be an efficient reliever of NASH development by modulating a broad spectrum of pathological mechanisms and could be applied in the future as an ingredient in supplements to promote liver health.

## Figures and Tables

**Table 1:** Sequence of primers used for RT-PCR.

Gene	Primer	Sequences (5′→3′)
*Fasn*	Forward Reverse	CAACCGGCTCTCTTTCTTCT CTTGGTAGGCATTCTGTAGTG
*Acaca*	Forward Reverse	AGCCAGAAG GGACAGTAGAA CTCAGCCAAGCGGATGTAAA
*Scd1*	Forward Reverse	GGCAGTTCTGAGGTGATTAGAG GTCTCTGGGAAG AGCAATGTAG
*Acyl*	Forward Reverse	CGGGAGGAAGCTGATGAATATG GTCAAGGTAGTGCCCAATGAA
*Srebf1*	Forward Reverse	GGACTTCCC ATCTGTTGTAAGG CAAGGGTTATGAGCCATGAGAT
*Pparg*	Forward Reverse	GCCTAAGTTTGAGTTTGCTGTG GCGGTCTCCACTGAGAATAATG
*Tnf* *a*	Forward Reverse	AATGGCCTCCCTCTCATCAGTT CCACTTGGTGGTTTGCTACGA
*Il6*	Forward Reverse	GAACAACGATGA TGCACTTGC TCCAGGTAGCTATGGTACTCC
*F4/80*	Forward Reverse	CGTCAGGTACGGGATGAATATAAG ATCTTGGAAGTGGATGGCATAG
*Mcp1*	Forward Reverse	CACTCACCTGCTGCTACTCA GCTTGGTGACAAAAACTACAGC
*Il12*	Forward Reverse	CTGGAACTACACAAGAACGAGAG GGCACA GGGTCATCATCAAA
*Il1* *b*	Forward Reverse	AGAGCCCATCCTCTGTGACTCA TGCTTGGGATCCACACTCTCCA
*Col1a1*	Forward Reverse	GAAACCCGAGGTATGCTTGA GTTGGGACAGTCCAGTTCTT
*Col3a1*	Forward Reverse	GGCTGCAAGATGGATGCTATAA GAATCTGTCCACCAGTGCTTAC
*Acta2*	Forward Reverse	CCATCATGCGTCTGGACTT GGCAGTAGTCACGAAGGAATAG
*Timp1*	Forward Reverse	GATTCAAGGCTGTGGGAAATG ACTCTTCACTGCGGTTCTG
*Tgf* *b*	Forward Reverse	CTTTAGGAAGGACCTGGGTTG GTGTGTCCAGGCTCCAAATA
*Il18*	Forward Reverse	CAGCCTGTGTTCGAGGATATG TCACAGCCAGTCCTCTTACT
*Pdgf* *a*	Forward Reverse	TCCAGCGACTCTTGGAGATA TCTCGGGCACATGGTTAATG
*18S rRNA*	Forward Reverse	GTAACCCGTTGAACCCCATT CCATCCAATCGGTAGTAGCG

## Data Availability

The datasets analyzed during the current study will not be shared publicly due to confidentiality reasons, but they are available from the corresponding author upon reasonable request.

## Supplementary Materials

The following supporting information can be downloaded at https://www.mdpi.com/article/10.3390/cimb46060351/s1, Figure S1: Composition profiles of YC-1102.
