# Supplementary material for: The Root Extract of Rosa multiflora Ameliorates Nonalcoholic Steatohepatitis Development via Blockade of De Novo Lipogenesis and Inflammation
Source: Curr Issues Mol Biol. 2024 Jun 12;46(6):5881–93. doi: 10.3390/cimb46060351 (PMC11202599; doi:10.3390/cimb46060351)
Supplement: Supplementary file 1 [file cimb-46-00351-s001.zip › cimb-3014045-supplementary.pdf]

Supplementary Materials

The root extract of *Rosa multiflora* ameliorates nonalcoholic steatohepatitis development via blockade of *de novo* lipogenesis and inflammation

Nam-Hee Kim[1*], Seung-Jin Lee[1*], Kyeong-Jin Lee[1], Ae Ri Song[3], Hyun-Je Park[3], Jong Soo Kang[3], Joo Young Cha[3#], and Yong-Hyun Han[1,2#]

[1]Laboratory of Pathology and Physiology, College of Pharmacy, [2]Multidimensional Genomics Research Center, Kangwon National University, Chuncheon 24341, South Korea

[3]Yuhan Care Co., Ltd., Yuhan Care R&D Center, Yongin-si 17084, Gyeonggi-do, South Korea

[*] These authors contributed equally to this work.

[#] Correspondence to: Joo Young Cha, Yuhan Care Co., Ltd., Yuhan Care R&D Center, , Tel: +82-31-303-0065, E-mail: jycha@yuhancare.com

Yong-Hyun Han, PhD, Laboratory of Pathology and Physiology, College of Pharmacy, Kangwon National University, Tel: +82-33-250-6908, E-mail: yhhan1015@kangwon.ac.kr

Supplementary Figure

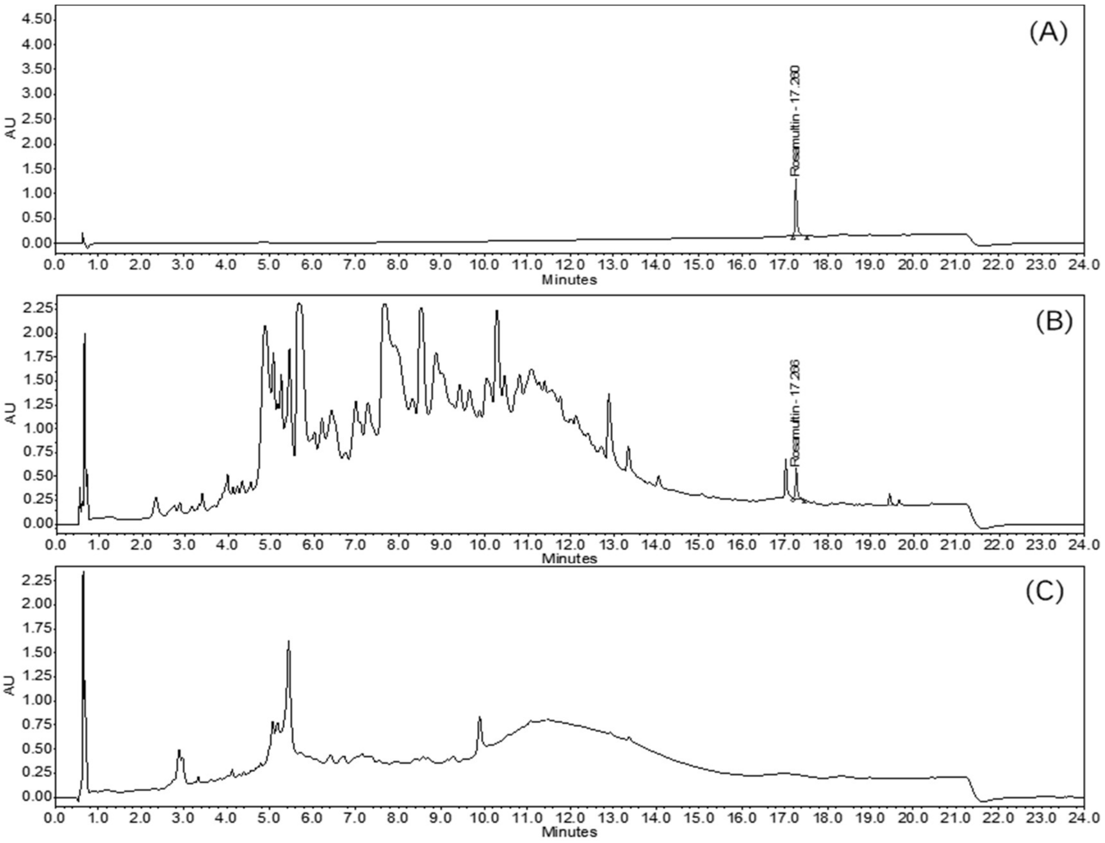

Supplementary Figure S1. Composition profiles of YC-1102

High-performance liquid chromatography (HPLC) results of (**A**) rosamultin as standard, (**B**) YC-1102 and (**C**) water extract of *Rosa multiflora* root
